# Dose Effects of Flubendiamide and Thiodicarb against *Spodoptera* Species Developing on Bt and Non-Bt Soybean

**DOI:** 10.3390/insects14090766

**Published:** 2023-09-14

**Authors:** Daniela N. Godoy, Venicius E. Pretto, Poliana G. de Almeida, Marlon A. G. Weschenfelder, Luiz F. Warpechowski, Renato J. Horikoshi, Samuel Martinelli, Graham P. Head, Oderlei Bernardi

**Affiliations:** 1 Department of Plant Protection, Federal University of Santa Maria (UFSM), Roraima Avenue 1000, Santa Maria 97105-900, Brazil; godoyufsm@yahoo.com (D.N.G.); veniciuspretto@outlook.com (V.E.P.); poolialmeida@gmail.com (P.G.d.A.); marlonweschenfelder6@gmail.com (M.A.G.W.); warpeagro@gmail.com (L.F.W.); 2 Regulatory Science, Bayer Crop Science, São Paulo 04779-900, Brazil; renato.horikoshi@bayer.com; 3 Regulatory Science, Bayer Crop Science, Chesterfield, MO 63017, USA; samuel.martinelli@bayer.com (S.M.); graham.head@bayer.com (G.P.H.)

**Keywords:** Bt soybean, chemical control, defoliator species, sublethal effects

## Abstract

**Simple Summary:**

Previous studies have reported that infestations by species of the genus *Spodoptera* (Lepidoptera: Noctuidae) have increased in Cry1Ac Bt soybean fields in Brazil, indicating that chemical insecticides will be needed to reduce crop damage. For that reason, we evaluated the dose effects of the insecticides flubendiamide and thiodicarb against *Spodoptera* species surviving on soybean expressing Cry1A.105, Cry2Ab2 and Cry1Ac Bt proteins; soybean expressing just Cry1Ac; and non-Bt soybean. Our results indicated that L1 and L2 larvae of *S. cosmioides*, *S. eridania* and *S. albula* surviving on Cry1A.105/Cry2Ab2/Cry1Ac and Cry1Ac soybean leaves sprayed with 50% or 100% of the field label dose of flubendiamide (70 mL/ha) or thiodicarb (400 g/ha) presented >86% mortality. Among the *Spodoptera* larvae surviving insecticide treatment, only some of the *S. frugiperda* larvae surviving on Cry1Ac and non-Bt soybean sprayed with 50% of the field label dose of flubendiamide or thiodicarb produced fertile adults. However, the surviving insects had a longer larval stage duration and the females laid fewer eggs, indicating substantial sublethal effects on the biological traits of this species.

**Abstract:**

An increase in *Spodoptera* species was reported in Bt soybean fields expressing Cry1Ac insecticidal proteins in Brazil, requiring additional management with chemical insecticides. Here, we evaluated the dose effects of flubendiamide and thiodicarb on *Spodoptera cosmioides* (Walker, 1858), *Spodoptera eridania* (Stoll, 1782), *Spodoptera albula* (Walker, 1857) and *Spodoptera frugiperda* (J. E. Smith, 1797) (Lepidoptera: Noctuidae) that survived on MON 87751 × MON 87708 × MON 87701 × MON 89788, expressing Cry1A.105, Cry2Ab2 and Cry1Ac; MON 87701 × MON 89788 soybean, expressing Cry1Ac; and non-Bt soybean. On unsprayed Cry1A.105/Cry2Ab2/Cry1Ac soybean, only *S. frugiperda* showed ~60% mortality after 10 d, whereas *S. cosmioides*, *S. eridania* and *S. albula* showed >81% mortality. The surviving larvae of all species on this Bt soybean showed >80% mortality when exposed to the field label dose of flubendiamide (70 mL/ha) or thiodicarb (400 g/ha) or at 50% of these doses. In contrast, all four species had <25% and <19% mortality on Cry1Ac and non-Bt soybean, respectively. The surviving *S. cosmioides*, *S. eridania* and *S. albula* on these soybean types presented >83% mortality after exposure to both dose levels of flubendiamide and thiodicarb. Some *S. frugiperda* larvae surviving on Cry1Ac and non-Bt soybean sprayed with a 50% dose of either insecticide developed into adults. However, the L1 larvae developing on Cry1Ac soybean leaves sprayed with flubendiamide and the L2 larvae on this soybean sprayed with thiodicarb had a prolonged immature stage, and the females displayed lower fecundity, which are likely to impact *S. frugiperda* population growth on soybean.

## 1. Introduction

The genus *Spodoptera* is composed of several species considered to be important agricultural pests, mainly in tropical and subtropical regions [1]. In Brazil, four species of the *Spodoptera* complex—*Spodoptera cosmioides* (Walker, 1858), *Spodoptera eridania* (Stoll, 1782), *Spodoptera albula* (Walker, 1857) and *Spodoptera frugiperda* (J. E. Smith, 1797) (Lepidoptera: Noctuidae)—attack soybean and other economically important crops such as maize and cotton [2]. Nonetheless, *Spodoptera* species are considered secondary pests of soybean in Brazil and other South American countries [3].

In 2013, the MON 87701 × MON 89788 soybean event (trade name Intacta RR2 PRO^®^), expressing the insecticidal protein Cry1Ac from *Bacillus thuringiensis* Berliner, became available to farmers in Brazil. This genetically modified (GM) soybean confers protection against *Anticarsia gemmatalis* (Hübner, 1818) (Lepidoptera: Erebidae) and against *Chrysodeixis includens* (Walker, [1858]), *Chloridea virescens* (Fabricius, 1781) and *Helicoverpa armigera* (Hübner, 1808) (Lepidoptera: Noctuidae) [4,5,6,7,8] and is cultivated on more than 36 million hectares per season in Brazil [9]. During the 2021/2022 season, the event MON 87751 × MON 87708 × MON 87701 × MON 89788 (trade name Intacta 2 Xtend^®^), expressing Cry1A.105, Cry2Ab2 and Cry1Ac proteins, was also released for commercial use in Brazil, extending the protection conferred by Bt soybean to the species *S. cosmioides* [10].

Previous reports have stated that Cry1A.105/Cry2Ab2/Cry1Ac and Cry1Ac soybean technologies had reduced lethality against *S. eridania* and *S. frugiperda* [5,8,10]. This can be explained by the naturally low susceptibility of these species to Bt proteins [5,11]. According to Horikoshi et al. [8], more than 98% of the insects sampled from the reproductive stages of Cry1Ac soybean in Brazil were *Spodoptera*, including *S. cosmioides*, *S. eridania* and *S. frugiperda*. Specifically for *S. frugiperda*, the resistance to Bt proteins expressed in maize affects the performance of Bt soybean technologies [12,13,14]. Regardless of their relatively low lethality against *Spodoptera* species, these Bt soybean technologies suppressed infestations of primary lepidopteran species in soybean in Brazil, enabling a decrease in the use of chemical insecticides and enhancing the natural biological control of other species [11,15].

Understanding both the lethal and the non-lethal effects of Bt proteins and insecticides on target and non-target lepidopteran pests is important for the best use of these control tactics in integrated pest management (IPM) programs. However, most studies have typically neglected the non-lethal effects (=sublethal effects) in favor of the more obvious lethal effects, which are measured through mortality analysis. Sublethal effects can be defined as those caused by low residual doses, which can exert effects on biological, physiological and biochemical processes as well as on insect development and reproduction [16,17,18]. For example, the sublethal effects of the insecticides and the Bt proteins extended the larval development and reduced the larval biomass, adult fecundity and population growth of several *Spodoptera* species [5,10,11,12,13,14,19,20,21]. Therefore, elucidating the overall effects (i.e., both lethal and sublethal) of Bt proteins and insecticides on pest species is essential for the design of robust IPM programs. Thus, we evaluated the dose effects of flubendiamide and thiodicarb against *S. cosmioides, S. eridania*, *S. albula* and *S. frugiperda* that survived on Bt and non-Bt soybean.

## 2. Materials and Methods

### 2.1. Collection and Rearing of Spodoptera Species

Brazilian populations of *S. cosmioides, S. eridania and S. albula* were collected from non-Bt soybean fields from the 2019/2020 to 2021/2022 seasons, whereas *S. frugiperda* were sampled from non-Bt maize during the 2021/2022 season. After collection, *Spodoptera* larvae were transported to the laboratory and reared on the artificial diet proposed by Greene et al. [22]. After two generations under laboratory conditions, *S. frugiperda* presented ~60% survival on Bt maize expressing Cry1 and Cry2 insecticidal proteins, indicating that the field-collected population had some degree of resistance to Bt proteins.

### 2.2. Soybean Plant Types

Seeds of GM soybean MON 87701 × MON 89788 × MON 87751 × MON 87708, which expresses Cry1A.105/Cry2Ab2/Cry1Ac insecticidal proteins (NEO590 I2X; GDM Genética do Brasil Ltda, Passo Fundo, RS, Brazil); GM soybean MON 87701 × MON 89788, expressing Cry1Ac protein (A5547 IPRO; Bayer Crop Science, São Paulo, SP, Brazil); and non-Bt soybean (NA 5909 RG; Nidera Sementes Ltda, São Paulo, SP, Brazil) were sown under field conditions at a density of 10 plants/m within each row and a spacing of 0.45 m between rows. Before bioassays, the plants were tested for the expression of Bt proteins using detection kits (QuickStix™; EnviroLogix, Portland, ME, USA).

### 2.3. Insecticides

The commercial products flubendiamide (Belt^®^ 480 g active ingredient (a.i.)/L)—ryanodine receptor modulator (IRAC MoA group 28)—and thiodicarb (Larvin 800 WG g a.i./kg)—acetylcholinesterase inhibitor (IRAC MoA sub-group 1A)—commonly applied to control lepidopteran soybean pests were provided by Bayer S.A. (São Paulo, SP, Brazil) and used to perform bioassays.

### 2.4. Bioassays

To measure the mortality of *Spodoptera* species on different soybean types, trifoliate leaves of Cry1A.105/Cry2Ab2/Cry1Ac soybean, Cry1Ac soybean and non-Bt soybean were excised from field-grown plants at the V_5–8_ growth stages [23]. In the laboratory, the trifoliate leaves were placed in 250 mL plastic pots over a gelled mixture of agar and water (2% agar), with a piece of filter paper separating the leaves from the agar–water layer. The leaves in each pot were infested with a total of 10 neonates (<24 h old), and the pots were placed in a climatic chamber at 25 ± 2 °C, 60 ± 10% RH and 14:10 h light:dark photoperiod. The leaves were replaced every 2 d, until all neonates had either died or progressed to L1 or L2.

The surviving L1 and L2 larvae were then transferred to leaves of the same soybean type that had been sprayed with the field label dose of flubendiamide (70 mL/ha) or thiodicarb (400 g/ha), sprayed with 50% of these doses, or unsprayed. To obtain these leaves, insecticides were applied to soybean plants cultivated under field conditions with a pressurized-CO_2_ backpack sprayer with a 2 m bar and a 0.5 m nozzle spacing (XR 110.02 fan-type nozzle tips; TeeJet Technologies Co., Glendale Heights, IL, USA), simulating a spray volume of 150 L/ha. Approximately 50 min after spraying, trifoliate leaves from the upper-third part of the plants were collected, and, in the laboratory, they were placed in 250 mL plastic pots as previously described. Unsprayed field-grown leaves of each soybean type were used as controls. Each pot was infested with 10 L1 or L2 larvae that had survived on unsprayed leaves of the same soybean type and then maintained in a climatic chamber under the same conditions described above. Larvae surviving 4 d of exposure to insecticide (or 4 d on unsprayed control leaves) were placed into 42-well plastic plates (1 larva/well) containing leaves collected from the same soybean type (Cry1A.105/Cry2Ab2/Cry1Ac, Cry1Ac or non-Bt) and treatment (insecticide and dose level) on which they previously survived. These leaves were collected from the plants initially sprayed with insecticide to simulate the residual effect of the insecticides under field conditions. The leaves were replaced every 2 d until larval mortality or pupation.

The experimental design was randomized with 7 replicates of 10 L1 or L2 larvae per insect species, soybean type and insecticide type/dose. The following lethal and sublethal effects were assessed: mortality at 10 d on Cry1A.105/Cry2Ab2/Cry1Ac, Cry1Ac and non-Bt soybean, survival from L1 and L2 stages to adult after exposure to sprayed and unsprayed leaves, developmental time from L1 or L2 stage to adult, number of eggs laid per female (fecundity) and egg-hatching percent (fertility). To evaluate fecundity and fertility, 4 to 10 couples from each treatment were paired in PVC cages (23 cm height × 10 cm diameter; one couple/cage) coated with white paper and closed at the top with sheer fabric. The adults were fed with a 10% honey/aqueous solution provided on cotton. Eggs were collected and counted every 2 d until the death of the female moth. To measure the egg-hatching percentage, 50 to 250 eggs from the second oviposition were obtained from each couple and placed in 100 mL plastic cups containing a piece of moistened filter paper.

### 2.5. Data Analysis

Mortality on Cry1A.105/Cry2Ab2/Cry1Ac, Cry1Ac and non-Bt soybean leaves sprayed with insecticides was corrected based on the mortality on unsprayed non-Bt soybean leaves according to Abbott’s formula [24]. Data on the mortality of all species and on the survival to adult phase, developmental time, fecundity and fertility of *S. frugiperda* were subjected to non-parametric analysis, and the mean differences were estimated through the Least Square Means Statement (LSMEANS option of PROC GLM) using a Tukey–Kramer test at *p* < 0.05 [25].

## 3. Results

### 3.1. Lethal Effects of Flubendiamide against Spodoptera Species Surviving on Different Soybean Types

**Figure 1:**
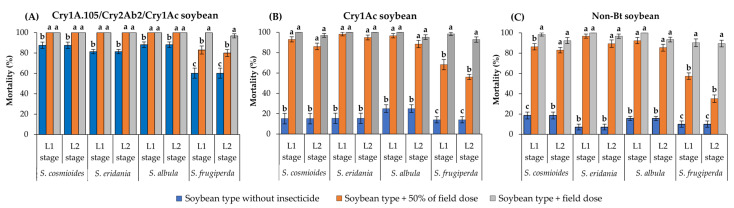
Lethality of three soybean types with and without flubendiamide spray against L1 and L2 larvae of *Spodoptera* species after 10 d of exposure. Within each group of bars (representing a given species and larval stage), values (±SE) with the same letter are not significantly different as determined by the LSMEANS statement using the Tukey–Kramer test at *p* > 0.05.

The mortality rate of *S. cosmioides*, *S. eridania* and *S. albula* ranged from 81.3 to 88.0% after 10 d on unsprayed Cry1A.105/Cry2Ab2/Cry1Ac soybean leaves (Figure 1A). However, on this same soybean type, the surviving L1 and L2 larvae presented significantly higher mortality (100%) on leaves sprayed with the field label dose of flubendiamide (70 mL/ha) or with 50% of this dose than on unsprayed leaves (F = 15.40; df = 2, 18; *p* < 0.0001 for *S. cosmioides*; F = 72.63; df = 2, 18; *p* < 0.0001 for *S. eridania*; F = 18.85; df = 2, 18; *p* < 0.0001 for *S. albula*) (Figure 1A). In contrast, *S. frugiperda* showed ~60% mortality after 10 d on unsprayed Cry1A.105/Cry2Ab2/Cry1Ac soybean leaves, but its mortality rate increased to more than 80% when the leaves were sprayed with either dose of flubendiamide (F = 30.31; df = 2, 18; *p* < 0.0001 for L1 stage; F = 24.87; df = 2, 18; *p* < 0.0001 for L2 stage) (Figure 1A).

On unsprayed Cry1Ac soybean, all *Spodoptera* species evaluated presented <25% mortality after 10 d (Figure 1B). The mortality rates of *S. cosmioides*, *S. eridania* and *S. albula* at the L1 and L2 stages developing on Cry1Ac soybean leaves were significantly higher on leaves sprayed with the field label dose of flubendiamide or with 50% of this dose (>86% mortality) than on unsprayed leaves of the same soybean type (F = 199.14–141.50; df = 2, 18; *p* < 0.0001 for *S. cosmioides*; F = 239.73–191.42; df = 2, 18; *p* < 0.0001 for *S. eridania*; F = 168.02–98.39; df = 2, 18; *p* < 0.0001 for *S. albula*) (Figure 1B). For *S. frugiperda*, the mortality rate was <14% on Cry1Ac soybean leaves but increased to >93% when the surviving L1 and L2 larvae were exposed to the same soybean type sprayed with the field label dose of flubendiamide (F = 145.76; df = 2, 18; *p* < 0.0001 for L1 stage; F = 207.77; df = 2, 18; *p* < 0.0001 for L2 stage) (Figure 1B).

Similar to previous results, on non-Bt soybean sprayed with either dose of flubendiamide, the mortality rates of the L1 and L2 larvae of *S. cosmioides*, *S. eridania* and *S. albula* were >83%, being significantly higher than on non-Bt soybean leaves without insecticide (<19% mortality) (F = 235.54–175.02; df = 2, 18; *p* < 0.0001 for *S. cosmioides*; F = 347.49–268.08; df = 2, 18; *p* < 0.0001 for *S. eridania*; F = 370.28–259.29; df = 2, 18; *p* < 0.0001 for *S. albula*) (Figure 1C). For *S. frugiperda*, the mortality rates of the L1 and L2 larval stages on non-Bt soybean leaves sprayed with the field label dose of flubendiamide (90% mortality) were significantly higher than on leaves sprayed with 50% of this dose (<57% mortality) and on unsprayed leaves (10% mortality) (F = 143.20; df = 2, 18; *p* < 0.0001 for L1 stage; F = 146.83; df = 2, 18; *p* < 0.0001 for L2 stage) (Figure 1C).

### 3.2. Lethal Effects of Thiodicarb against Spodoptera Species Surviving on Different Soybean Types

**Figure 2:**
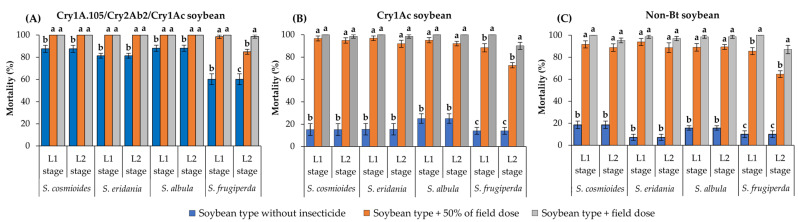
Lethality of soybean types with and without thiodicarb spray against L1 or L2 larvae of *Spodoptera* species after 10 d of exposure. Group of bars (±SE) with the same letter in each larval stage are not significantly different as determined by the LSMEANS statement using Tukey–Kramer test at *p* > 0.05.

The mortality rates of *S. cosmioides*, *S. eridania* and *S. albula* on unsprayed Cry1A.105/Cry2Ab2/Cry1Ac soybean leaves were >81% after 10 d of exposure (Figure 2A). However, the surviving L1 and L2 larvae of these species presented significantly higher mortality (100%) when exposed to leaves of the same soybean type sprayed with the field label dose of thiodicarb (400 g/ha) or with 50% of this dose (F = 15.40; df = 2, 18; *p* < 0.0001 for *S. cosmioides*; F = 72.63; df = 2, 18; *p* < 0.0001 for *S. eridania*; F = 18.85; df = 2, 18; *p* < 0.0001 for *S. albula*) (Figure 2A). *Spodoptera frugiperda* had ~60% mortality on unsprayed Cry1A.105/Cry2Ab2/Cry1Ac soybean, but the surviving L1 larvae exposed to the same soybean type sprayed with either dose of thiodicarb showed >98% mortality (F = 57.08; df = 2, 18; *p* < 0.0001). In contrast, the L2 larvae presented lower mortality on leaves sprayed with 50% of the thiodicarb field label dose (83.9% mortality) than on those sprayed with the field label dose (98.7% mortality) (F = 35.57; df = 2, 18; *p* < 0.0001) (Figure 2A).

The mortality rates of *S. cosmioides*, *S. eridania* and *S. albula* were <25% on unsprayed Cry1Ac soybean leaves after 10 d of exposure (Figure 2B). However, the surviving L1 and L2 larvae of these species presented mortality ranging from 91.8% to 100% on Cry1Ac soybean leaves sprayed with either dose of thiodicarb, differing significantly from the mortality on unsprayed leaves of the same soybean type (F = 217.16–186.78; df = 2, 18; *p* < 0.0001 for *S. cosmioides*; F = 228.06–164.26; df = 2, 18; *p* <0.0001 for *S. eridania*; F = 161.03–151.13; df = 2, 18; *p* < 0.0001 for *S. albula*) (Figure 2B). Similarly, the mortality of the L1 and L2 stages of *S. frugiperda* on unsprayed Cry1Ac soybean leaves was <15%, but these larval stages presented >72.6% mortality on Cry1Ac leaves sprayed with either dose of thiodicarb (F = 143.20; df = 2, 18; *p* < 0.0001 for L1 stage; F = 146.83; df = 2, 18; *p* < 0.0001 for L2 stage) (Figure 2B).

With the exception of the L2 stage of *S. frugiperda* developing on non-Bt soybean sprayed with 50% of the field label dose of thiodicarb (64.6% mortality), all other *Spodoptera* species presented >88.6% mortality when exposed to either dose of thiodicarb, differing significantly from those that developed on unsprayed non-Bt leaves (<19% mortality) (F = 272.14–190.82; df = 2, 18; *p* < 0.0001 for *S. cosmioides*; F = 380.34–236.36; df = 2, 18; *p* < 0.0001 for *S. eridania*; F = 344.46–361.43; df = 2, 18; *p* < 0.0001 for *S. albula*) (Figure 2C).

### 3.3. Sublethal Effects of Insecticides against Spodoptera Species Surviving on Different Soybean Types

#### 3.3.1. Effects on Development

It is important to highlight that no L1 and L2 larvae of *S. cosmioides*, *S. eridania* or *S. albula* initially surviving on unsprayed Cry1A.105/Cry2Ab2/Cry1Ac soybean leaves developed to adulthood on either sprayed or unsprayed leaves, making it impossible to measure the sublethal effects of insecticides on these species. Only *S. frugiperda* larvae developing on Cry1A.105/Cry2Ab2/Cry1Ac soybean leaves without insecticide developed into adults (<63%).

**Figure 3:**
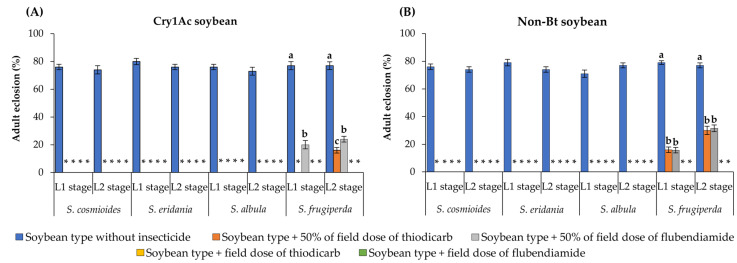
Adult eclosion (%) of *Spodoptera* species exposed to soybean types with and without insecticide spray. Within each group of bars (representing a given species and larval stage), values (±SE) with the same letter are not significantly different as determined by the LSMEANS statement using the Tukey–Kramer test at *p* > 0.05. An asterisk (*) indicates that no insects survived until adulthood.

On Cry1Ac and non-Bt soybeans, none of the *Spodoptera* larvae developed to adulthood on insecticide-sprayed leaves except for some *S. frugiperda* larvae exposed to leaves sprayed with 50% of the field label dose of flubendiamide or thiodicarb (Figure 3A,B). On Cry1Ac soybean leaves, <24% of *S. frugiperda* larvae developed to adulthood after exposure to 50% of the field label dose of flubendiamide or thiodicarb, differing significantly from the frequency developing on leaves sprayed with the field label dose (no survival) or on unsprayed leaves (77% developed into adults) (F = 184.62–282.08; df = 1, 18; *p* < 0.0001) (Figure 3A). Similar results were obtained on non-Bt soybean: <32% of larvae developed into adults on leaves sprayed with 50% of the field label dose of flubendiamide or thiodicarb, whereas on unsprayed leaves, survival to adulthood was ~78% (F = 296.20–119.32; df = 2, 18; *p* < 0.0001). When the surviving *S. frugiperda* larvae were exposed to non-Bt soybean leaves sprayed with field label doses of either insecticide, none developed to adulthood (Figure 3B).

The developmental time to adult eclosion of the L1 larvae of *S. frugiperda* surviving on leaves of Cry1Ac soybean sprayed with 50% of the field label dose of flubendiamide was significantly longer than that of the larvae developing on the same soybean type without insecticide (F = 12.06; df = 1, 12; *p* = 0.0047) (Table 1). Similarly, a longer developmental time to adult eclosion was observed for L1 larvae developing on non-Bt leaves sprayed with 50% of the field label dose of thiodicarb than for those on unsprayed leaves (F = 65.58; df = 1, 12; *p* = < 0.0001). In contrast, the L1 larvae of *S. frugiperda* on non-Bt soybean had similar developmental times on leaves with 50% of the flubendiamide dose and on unsprayed leaves (F = 4.43; df = 1, 12; *p* = 0.0571). No L1 larvae survived to adulthood on Cry1Ac soybean leaves sprayed with 50% of the thiodicarb field label dose (Table 1).

On the other hand, the immature stage of the L2 larvae was ~6 d longer on Cry1Ac soybean leaves sprayed with 50% of the thiodicarb field label dose than on unsprayed leaves (F = 175.84; df = 1, 12; *p* < 0.0001) (Table 1). In contrast, the L2 larvae of *S. frugiperda* on Cry1Ac soybean leaves sprayed with 50% of the field label dose of flubendiamide or unsprayed leaves had similar developmental times to adult eclosion (F = 2.48; df = 1, 12; *p* = 0.1410). The development times were also similar between L2 larvae reared on non-Bt soybean sprayed with 50% of the field label dose of flubendiamide and those on unsprayed leaves (F = 1.07; df = 1, 12; *p* = 0.3213). However, L2 larvae reared on non-Bt soybean sprayed with 50% of the thiodicarb dose had a 2 d longer developmental time until adult eclosion than those reared on unsprayed leaves (F = 22.56; df = 1, 12; *p* = 0.0005) (Table 1). In general, *S. frugiperda* larvae developing on Bt and non-Bt soybean leaves sprayed with non-lethal quantities of flubendiamide or thiodicarb showed prolonged developmental time to adult eclosion.

#### 3.3.2. Effects on Reproduction

The egg-laying capacity of *S. frugiperda* females decreased when the larvae were exposed to 50% of the field label doses of flubendiamide or thiodicarb applied to Cry1Ac and non-Bt soybean (Table 2). The adult females obtained from larvae that had developed on Cry1Ac soybean leaves sprayed with 50% of the field label dose of flubendiamide (from L1 and L2 larvae) or thiodicarb (only from L2 larvae) laid 25% fewer eggs than those from larvae fed on Cry1Ac soybean without insecticide (F = 5.91; df = 1, 12; *p* = 0.0316 for L1 stage; F = 6.27; df = 2, 18; *p* = 0.0086 for L2 stage). Similarly, the females from L1 or L2 larvae reared on non-Bt soybean sprayed with a 50% dose of flubendiamide or thiodicarb also had reduced fecundity (24–31% fewer eggs laid) compared with females from larvae raised on non-Bt soybean without insecticide (F = 9.33; df = 2, 15; *p* = 0.0023 for L1 stage; F = 19.71; df = 2, 20; *p* < 0.0001 for L2 stage) (Table 2). However, regardless of the soybean type, the percentage of eggs hatching did not differ for females obtained from larvae developing on sprayed versus unsprayed soybean leaves (>82.6% eggs hatching) (*p* > 0.0503) (Table 2).

## 4. Discussion

The GM soybean event MON 87701 × MON 89788 × MON 87751 × MON 87708, which expresses Cry1A.105/Cry2Ab2/Cry1Ac insecticidal proteins, showed high lethality against *S. cosmioides*, *S. albula* and *S. eridania* but had relative low lethality against *S. frugiperda* after 10 d of exposure. Except for *S. frugiperda*, no larvae of the tested *Spodoptera* species developed into adults on this soybean type. Results from previous studies also showed that no larvae of S. cosmioides, S. albula or S. eridania were able to develop to the adult stage on Cry1A.105/Cry2Ab2/Cry1Ac soybean [10]. Nonetheless, none of the L1 and L2 larvae of *Spodoptera* species surviving on Cry1A.105/Cry2Ab2/Cry1Ac soybean for 10 d (~40% for *S. frugiperda* and <20% for other species tested) survived until the adult stage when exposed to leaves sprayed with flubendiamide or thiodicarb at the field label dose or 50% of this dose.

The survival of *S. frugiperda* until adult stage on Cry1A.105/Cry2Ab2/Cry1Ac soybean can be explained by the cross-resistance with Bt proteins expressed in maize and the high frequency of Cry protein resistance alleles in Brazilian populations of this species [15,26]. This cross-resistance occurs due to the similarity of Cry1 proteins expressed in maize and soybean, which have 60 to 90% similarity in their amino acid sequences [27]. Additionally, the Cry1 proteins also share the same binding site in the midgut of the *S. frugiperda* [28,29] whereas the Cry2Ab2 protein does not have high toxicity against *S. frugiperda* [30]. These characteristics negatively affect the performance of further Bt plants, including cotton and other types of Bt soybean, against this species [10,14]. Therefore, in growing areas cultivated with Cry1A.105/Cry2Ab2/Cry1Ac soybean, complementary chemical control is needed only when the number of *S. frugiperda* exceeds the economic threshold level (20 large larvae (≥1.5 cm) per sample cloth in 1 m of soybean row, 30% defoliation at the vegetative stage or 15% defoliation at the reproductive stage) [31]. In contrast to the results with Cry1A.105/Cry2Ab2/Cry1Ac soybean, MON 87701 × MON 89788 (Cry1Ac soybean) presented reduced lethality against all *Spodoptera* species, with more than 73% developing into adults. Previous studies also reported low lethality of this Bt soybean against *Spodoptera* species [5,8,10]. Similar survival until the adult stage was also observed when *Spodoptera* species were grown on non-Bt soybean leaves (>71% developed into adults). However, *S. cosmioides*, *S. eridania* and *S. albula* larvae surviving on Cry1Ac or non-Bt soybean did not produce adults when exposed to soybean leaves sprayed with both doses of flubendiamide or thiodicarb. The *S. frugiperda* larvae that survived on these soybean types when exposed to a 50% dose of either insecticide could produce adults but not when exposed to the full field label doses. These results indicate that, if outbreaks of these species are detected in Cry1Ac soybean fields, insecticide application is indicated when action thresholds are reached, since these species have a high defoliation capacity (mainly *S. cosmioides*) and also attack flowers and pods during the reproductive stages, reducing soybean yield [31,32].

In addition to lethal effects, the exposure of insects to sublethal concentrations of insecticides can affect insect behavior, physiology and ecology [18]. In our study, the sublethal effects of the Bt proteins and insecticides on *S. frugiperda* developing on soybean leaves (Bt and non-Bt) sprayed with 50% of the field label dose of flubendiamide or thiodicarb were evident: these insects had an extended larval stage and the females had lower fecundity than insects that had developed on the same soybean leaf type without insecticide. Previous studies also reported the substantial sublethal effects of chlorantraniliprole (same mode of action of flubendiamide), which reduced the number of eggs laid by *S. frugiperda* [33]. Chlorantraniliprole also prolonged the larval, pupal and generation times of *Spodoptera exigua* (Hübner, 1808) (Lepidoptera: Noctuidae), and it increased the life cycle time and reduced the fecundity of *S. cosmioides* [34,35], whereas flubendiamide reduced the fecundity and fertility of *Spodoptera litura* (Fabricius, 1775) (Lepidoptera: Noctuidae) [36,37]. Studies also reported relevant sublethal effects of Bt proteins expressed on soybean, reducing the larval biomass, adult fecundity and population growth potential of several Spodoptera species, including *S. frugiperda* [5,10,11,12,13,14]. In summary, the *Spodoptera* species surviving on Bt and non-Bt soybean exposed to non-lethal quantities of flubendiamide or thiodicarb had prolonged immature stages and decreased female reproductive performance.

Our results indicated that the combination of control tactics is an important component of IPM, and the use of Bt plants can help manage lepidopteran pests that are not targeted by the Bt technologies but exhibit sublethal effects from these technologies that could enhance the level of control of chemical insecticides. Alternatively, non-insecticidal management strategies such as natural enemies can also increase the control of *Spodoptera* larvae that survive in Bt soybean, since larval development is delayed due to the non-lethal effects of the Bt proteins. From a pest-control perspective, it is also important that the Bt soybean technologies are grown according to refuge requirements (at least 20% of structured refuge cultivated with non-Bt soybean) and when insecticides are applied, rotation of modes of action. Therefore, integrating multiple control tactics can prolong the durability of Bt technologies and chemical insecticides, mainly in an intensive agricultural system, such as that in Brazil, where resistance is already widespread.

## 5. Conclusions

In this study, we documented the lethal and sublethal effects of flubendiamide and thiodicarb on *Spodoptera* species surviving on different Bt and non-Bt soybean types. The lethal effects included the following: (i) after 10 d of exposure, Bt soybean expressing Cry1A.105/Cry2Ab2/Cry1Ac insecticidal proteins presented high lethality against the larvae of *S. cosmioides*, *S. albula* and *S. eridania* but relatively low lethality against *S. frugiperda*; (ii) Bt soybean expressing only Cry1Ac insecticidal protein had low lethality against the larvae of all *Spodoptera* species tested; (iii) none of the *Spodoptera* larvae exposed to leaves sprayed with the field label dose of flubendiamide or thiodicarb survived to adulthood, regardless of the soybean plant type; and (iv) among the insecticide-exposed larvae, only *S. frugiperda* that survived on Cry1Ac and non-Bt soybean sprayed with 50% of the field label dose of flubendiamide or thiodicarb survived to adulthood. The sublethal effects on *S. frugiperda* of flubendiamide or thiodicarb applied to Cry1Ac and non-Bt soybean included prolonged immature stage and lower female fecundity, suggesting population suppression of this pest. In summary, the lethal and sublethal effects here reported can help to optimize the management of *Spodoptera* species on Bt and non-Bt soybean fields in Brazil and other countries.

## Figures and Tables

**Table 1:** Developmental time to adult eclosion of *S. frugiperda* fed on Cry1Ac and non-Bt soybean sprayed with flubendiamide or thiodicarb (50% dose) compared to unsprayed.

Cry1Ac Soybean ^1^				Non-Bt Soybean ^1^		
Stage	Bt Soybean (Unsprayed)	Bt Soybean + 50% of Flubendiamide Dose	Bt Soybean + 50% of Thiodicarb Dose	Non-Bt Soybean (Unsprayed)	Non-Bt + 50% of Flubendiamide Dose	Non-Bt + 50% of Thiodicarb Dose
L1	25.6 ± 0.10 b	29.3 ± 0.60 a	*	27.1 ± 0.15 b	28.2 ± 0.42 b	30.6 ± 0.61 a
L2	22.3 ± 0.16 b	23.0 ± 0.31 b	28.2 ± 0.42 a	22.6 ± 0.18 b	22.2 ± 0.29 b	24.3 ± 0.25 a

^1^ Means (±SE) followed by the same letter within each soybean type and larval stage are not significantly different as determined by the LSMEANS statement using the Tukey–Kramer test at *p* > 0.05. An asterisk (*) indicates that no insects survived until adulthood.

**Table 2:** Reproductive parameters of *S. frugiperda* developing on Cry1Ac and non-Bt soybean sprayed with flubendiamide or thiodicarb (50% dose) compared to unsprayed.

Cry1Ac Soybean ^1^				Non-Bt Soybean ^1^		
Stage	Bt Soybean(Unsprayed)	Bt Soybean + 50% of Flubendiamide Dose	Bt Soybean + 50% of Thiodicarb Dose	Non-Bt Soybean (Unsprayed)	Non-Bt + 50% of Flubendiamide Dose	Non-Bt + 50% of Thiodicarb Dose
Eggs/female/day						
L1	95.2 ± 4.5 a	76.1 ± 5.0 b	*	101.8 ± 5.2 a	77.4 ± 1.8 b	70.3 ± 5.9 b
L2	104.3 ± 3.3 a	92.0 ± 5.8 ab	78.4 ± 8.0 b	118.9 ± 3.9 a	87.1 ± 2.5 b	81.6 ± 7.7 b
Eggs hatching (%)						
L1	90.3 ± 1.3 a	87.9 ± 0.7 a	*	91.9 ± 1.4 a	91.0 ± 1.1 a	82.7 ± 5.2 a
L2	92.7 ± 1.3 a	91.8 ± 0.5 a	89.7 ± 1.0 a	93.9 ± 1.2 a	89.5 ± 1.7 a	

^1^ Means (±SE) followed by the same letter within each soybean type and larval stage are not significantly different as determined by the LSMEANS statement using the Tukey–Kramer test at p > 0.05. An asterisk (*) indicates that no insects survived until adulthood.

## Data Availability

The data presented in this study are available in this article.
